# Analysis of peri-implant bone tissue between hydrophilic and rough implant surfaces in spontaneously hypertensive rats treated with losartan

**DOI:** 10.1590/1678-7757-2023-0374

**Published:** 2024-06-10

**Authors:** Jaqueline Silva dos SANTOS, Gabriel MULINARI-SANTOS, Fábio Roberto DE SOUZA BATISTA, Pedro Henrique Silva GOMES-FERREIRA, Letícia Pitol PALIN, Cristina ANTONIALI, Roberta OKAMOTO

**Affiliations:** 1 Universidade Estadual Paulista “Júlio de Mesquita Filho” Faculdade de Odontologia de Araçatuba Departamento de Ciências Básicas Araçatuba Brasil Universidade Estadual Paulista “Júlio de Mesquita Filho”, Faculdade de Odontologia de Araçatuba, Departamento de Ciências Básicas, Araçatuba, Brasil.; 2 Universidade Estadual Paulista “Júlio de Mesquita Filho” Faculdade de Odontologia de Araçatuba Departamento de Diagnóstico e Cirurgia Araçatuba Brasil Universidade Estadual Paulista “Júlio de Mesquita Filho”, Faculdade de Odontologia de Araçatuba, Departamento de Diagnóstico e Cirurgia, Araçatuba, Brasil.

**Keywords:** Dental implants, Bone remodeling, Hypertension, Losartan, SHR rat

## Abstract

Hypertensive individuals present alterations in their calcium metabolism that consequently decrease the concentration of this mineral in the bone tissue.

**Objectives:**

to evaluate the morphological and functional characteristics of the peri-implant bone tissue that was formed during the healing process by the placement implants using two different surface treatments: hydrophilic Acqua™ (ACQ) and rough NeoPoros™ (NEO), in spontaneously hypertensive (SHR) and normotensive rats (Wistar) whether or not treated with losartan.

**Methodology:**

In total, 96 male rats (48 Wistar and 48 SHR) were divided into eight subgroups: absolute control rough (COA NEO), absolute control hydrophilic (COA ACQ), losartan control rough (COL NEO), losartan control hydrophilic (COL ACQ), SHR absolute rough (SHR NEO), SHR absolute hydrophilic (SHR ACQ), SHR losartan rough (SHRL NEO), and SHR losartan hydrophilic (SHRL ACQ). The rats medicated with losartan received daily doses of the medication. NeoPoros™ and Acqua™ implants were installed in the tibiae of the rats. After 14 and 42 days of the surgery, the fluorochromes calcein and alizarin were injected in the rats. The animals were euthanized 67 days after treatment. The collected samples were analyzed by immunohistochemistry, biomechanics, microcomputerized tomography, and laser confocal scanning microscopy analysis.

**Results:**

The osteocalcin (OC) and vascular endothelium growth factor (VEGF) proteins had moderate expression in the SHRL ACQ subgroup. The same subgroup also had the highest implant removal torque. Regarding microarchitectural characteristics, a greater number of trabeculae was noted in the control animals that were treated with losartan. In the bone mineralization activity, it was observed that the Acqua™ surface triggered higher values of MAR (mineral apposition rate) in the COA, COL, and SHRL groups (p<0.05).

**Conclusion:**

the two implant surface types showed similar responses regarding the characteristics of the peri-implant bone tissue, even though the ACQ surface seems to improve the early stages of osseointegration.

## Introduction

Arterial hypertension is a systemic alteration that affects a large part of the population, impacting one in three young adults.^1^ In addition to cardiovascular changes, hypertensive individuals have lower plasma concentrations of calcium and vitamin D, which are essential precursors for maintaining bone metabolism.^2,3^

The renin-angiotensin system is an important regulator of blood pressure. It directly acts in the etiopathogenesis of this condition (arterial hypertension). When unbalanced, renin-angiotensin increases the values of systolic and diastolic pressure due to effects on peripheral arterial resistance (related to diastolic pressure) and fluid retention. Many substances that work on the regulation of this system have been used for the treatment of hypertension and among them, losartan stands out.^4-6^ Losartan is a medication commonly consumed by hypertensive individuals due to its effectiveness and because its side effects are minimal. It works by blocking angiotensin II AT1 receptors, decreasing vasoconstriction and aldosterone secretion and reducing altered blood pressure to normal levels.^7,8^ This medication indirectly intervenes in the bone metabolism due to its effect on the OPG/RANKL system. Studies have shown the positive effect of losartan regarding bone-implant contact, alveolar repair, and decreased periodontal bone loss and orthodontic movement in spontaneously hypertensive rats (SHR).^9-13^

Due to the demand for aesthetic and functional rehabilitation by dental implants, a resource used to accelerate and optimize the osseointegration process is the surface treatments on the implants.^14,15^ Among the different types of treatments used, surfaces blasted by blasting abrasive and acid conditioning kept in NaCl solution (hydrophilic) are broadly used in the clinical protocol. The physicochemical alterations proposed by this type of treatment improve the wettability and hydrophilicity of the implant, enhancing adhesion and holding clot blood, favoring the beginning of the osseointegration process.^16-18^

According to information provided by the manufacturer (Neodent^®^), Acqua™ implants incorporate a hydrophilic physicochemical surface technology conducive to cellular differentiation on the titanium surface treated with abrasive blasting and acid conditioning. This approach results in no topographic or roughness alterations but rather in a chemical transformation of the titanium oxide layer. In contrast, NeoPoros™ surface implants (Neodent^®^), which undergo prior abrasive blasting and acid conditioning, exhibit changes in their surface roughness due to the formation of microcavities induced by oxide particles.^19^The association between this different type of surface treatment and losartan could synergistically act on the peri-implant repair process impaired by hypertension.

Spontaneously hypertensive Wistar rats (SHR) are experimental models established in the scientific literature to evaluate the effects of angiotensin II receptor antagonist drugs against adverse bone metabolic effects resulting from arterial hypertension.^20-22^ That said, this study aimed to evaluate the osseointegration of implants with different surface treatments (NeoPoros™ and Acqua™) that were installed in the tibiae of spontaneously hypertensive rats (SHR) treated with losartan by removal torque, microcomputerized tomography, and bone dynamics by fluorochromes.

## Methodology

### Study design

After the study was approved by the Ethics Committee in the Use of Animals (CEUA) (2016/00404) at the Araçatuba Dental School, São Paulo State University (UNESP), 96 male rats (*Rattus norvegicus*, albinus) (n=64) were used. This study used 48 normotensive rats (Wistar) (n=48) and 48 spontaneously hypertensive rats (SHR) (n=48) aged 16 weeks of life. Each animal weighted approximately 350 grams. The animals were kept in cages at 22° C (± 2° C), with controlled light cycles (12 hours light and 12 hours dark), a balanced diet (Ração Mogiana Alimentos SA, Campinas, SP, Brazil), and a controlled amount of water.

The groups were randomized by a computer-generated list, and all analyses were evaluated via calibration and blinded examination. The rats were divided into four groups according to whether the drug was or not used: COA (absolute control, n=24), COL (losartan control, n=24), SHR (absolute SHR, n=24), and SHRL (SHRL losartan, n=24). After the surgical procedure, two subgroups were inserted in each of the groups previously named according to the type of surface treatment of the implants (NeoPoros™ and Acqua™), containing eight (n=8) animals per subgroup: absolute control rough (COA NEO), absolute control hydrophilic (COA ACQ), losartan control rough (COL NEO), losartan control hydrophilic (COL ACQ), SHR absolute rough (SHR NEO), SHR absolute hydrophilic (SHR ACQ), SHR losartan rough (SHRL NEO), and SHR losartan hydrophilic (SHRL ACQ).

The eight animals of each subgroup were used in all analyses. In total, four animals were employed in the immunohistochemical analysis; the same animals were used in the biomechanical analysis. Another four animals were employed in computerized microtomography and subsequently in laser confocal microscopy.

### Losartan administration and blood pressure

Losartan (Biosintética, São Paulo, Brazil) was applied daily at 30 mg in drinking water per kilogram body weight.^20-22^ The treatment started seven days prior to implant placement. Systolic blood pressure was checked by tail cuff indirect plethysmography using Physiograph^®^, MK-III-S (Narco Bio-systems, Houston, TX), adapted for measurements in rats.^20-22^ The medication failed to alter the blood pressure of normotensive rats and controlled the blood pressure of the hypertensive ones. Losartan was given to the animals and tail cuff indirect plethysmography was performed every day until the animals were euthanized.

### Installation of implants

Overall, seven days after the initial treatment, the rats (n=96) fasted for eight hours prior to the surgery to then be sedated with a combination of 50 mg/kg of ketamine (Vetaset^®^ - Fort Dodge Animal Health Ltda.; Campinas, SP, Brasil) and 5 mg/kg of xylazine hydrochloride (Dopaser - Laboratórios Calier do Brasil Ltda.; Osasco, SP, Brazil), given via intramuscular injection. Trichotomy was performed, followed by topical antisepsis using povidone-iodine topical (10% PVP-I, Degeneração de Riodeine Soft Derma, Rioquímica, São José do Rio Preto, SP, Brasil) in the region of the tibial metaphyses of each animal. An incision of 3 cm was done with soft tissue avulsion up to the exposure of tibial metaphysis. After the incision, milling was performed with a spiral milling cutter with 1.5 mm in diameter mounted on an electric motor (BLM 600^®^; Driller, São Paulo, SP, Brazil) at a speed of 1.000 rpm and under irrigation with a 0.9% saline solution (Fisiológico^®^, Biosintética Ltda., Ribeirão Preto, SP, Brazil). Installation was manually conducted with a digital key. Grade IV titanium screws of 1.5 mm in diameter and 3.5 mm length were implanted in each tibia. The left tibiae of the rats received sandblasted implants - NeoPoros™ (Neodent^®^, Curitiba, PR, Brazil), whereas the right tibiae received hydrophilic surface implants - Acqua™ (Neodent^®^, Curitiba, PR, Brazil). The deep wounds were closed with resorbable sutures (Poliglactina 910 - Vicryl 4.0, Ethicon, Johnson & Johnson, São José dos Campos, SP, Brazil). Monofilament sutures (Nylon 5.0, Ethicon, Johnson & Johnson, São José dos Campos, SP, Brazil) were used close to the external wounds. Each animal received an intramuscular injection of pentabiotic (0,1 ml/kg; Fort Dodge Saúde Animal Ltda.; Campinas, SP, Brazil) and sodic dipyrone (1 mg/kg; Ariston, Indústrias Químicas e Farmacêuticas Ltda.; São Paulo, SP, Brazil) in the immediate postoperative period.^21,23^

### Fluorochrome application

Overall, 14 days postoperative, fluorochrome calcein was given to four animals in each group via intramuscular injection (20 mg/kg). After 28 days (42 days after the implants were installed), the alizarin red fluorochrome (20 mg/kg) was injected into the animals.^21,23,24^Both fluorochromes indicate, respectively, the deposition of old bone and newly formed bone.

### Euthanasia

The animals (n=96) were subjected to euthanasia by an anesthetic overdose administered intramuscularly (150 mg/kg, Tiopental Cristália Ltda.; Itapira, SP, Brazil) 60 days after the installation of implants.^21,23^

### Immunohistochemical analysis

For this analysis, the samples for each group (n=4) were then fixed in 10% formaldehyde (Reagentes Analíticos, Dinâmica Odonto‐Hospitalar Ltda.; Catanduva, SP, Brazil) for 48 hours, washed off in running water for 24 hours, and underwent decalcification in EDTA 10% for 20 weeks. The samples were then dehydrated using a gradual and increasing alcohol concentration sequence. Subsequently, they were diaphanized with xylol, embedded in paraffin to obtain slices of 5 μm thickness using a microtome (Leica Biosystems – RM2125 RTS, Heerbrugg, Switzerland), and inserted on glass mounted slides. The immunohistochemical process started by deparaffinating the samples by washing the slides and incubating them in a CitriSolv bath (Decon Labs, Inc.; King of Prussia, PA, USA), followed by incubating the same slides in decreasing concentrations of alcohol baths, and finalizing this part of the process by hydrating the cuts immersed in PBS (Phosphate Buffered Salin 0.01M). Endogenous peroxidase activity was inhibited with hydrogen peroxide, followed by having the slides undergo antigenic recovery with a citrate phosphate buffer solution (pH 6.0) in humid heat. Skim milk was used to block endogenous biotin. As a method of blocking nonspecific markings, the primary antibody was prepared in a citrate phosphate buffer solution and bovine albumin 1%. Primary antibodies (polyclonal antibodies produced in goats) were put against osteocalcin (OC) (SC18319, Santa Cruz Biotechnology, Inc. 10410 Finnell Street, Dallas, TX, USA) to characterize the degree of mineralization and advancement of maturity of the bone tissue, osteoprotegerin (OPG) (SC21038, Santa Cruz Biotechnology, Inc. 10410 Finnell Street, Dallas, TX, USA), and the receptor activator of nuclear factor kappa-Β ligand, (RANKL) (SC7627, Santa Cruz Biotechnology Inc. 10410 Finnell Street, Dallas, TX, USA) to evaluate the turnover at the bone-implant interface and then, against the vascular endothelium growth factor (VEGF) (SC7269, Santa Cruz Biotechnology, Inc. 10410 Finnell Street, Dallas, TX, USA) to show evidence of the blood supply in the region of interest. Biotinylated rabbit anti-goat was used as secondary antibody (Histofine^®^ - Immunohistochemical staining reagent, San Diego, CA, USA). The reaction was amplified by incubation in avidin and biotin and the reaction was shown by using diaminobenzidine (Dako Omnis - Agilent Dako, Santa Clara, CA, USA). At the end of the immunohistochemical analysis, the slides were counterstained with Harris’ hematoxylin (Merck KGaA, Burlington, MA, USA). The slides were then dehydrated by using xylol, and the glasses coverslips were mounted into the slides. The images were captured by optical microscope (Leica Microsystems DMLB, Heerbrugg, Switzerland). For each of the used antibodies, the qualitative analysis of protein expression was assessed by assigning different scores according to the number of cells and the immunostained extracellular matrix area during the osseointegration process. Immunolabeling was characterized for different proteins that showed the process of bone formation and mineralization, with scores of 0 (absence of labeling), 1 (light labeling), 2 (moderate labeling), and 3 (intense labeling).^22-25^ A unique blinded and calibrated evaluator performed the immunohistochemical analysis. In total, four sections were used for each protein per group.

### Biomechanical analysis

The samples (n=4, per group) were biomechanically evaluated. The short connection set for contra-angle (Neodent, Curitiba, PR, Brazil) and the torque wrench were positioned on the implant, and counterclockwise movement was applied, generating reverse torque until the interface of implant was released from the bone. The device registered the maximum torque peak in Newton per centimeter (N.cm).^21,25,26^

### Microcomputerized tomography

Some parts of the tibiae (n=4 per group) that were collected were fixed in 10% formaldehyde (Reagentes Analíticos^®^, Dinâmica Odonto‐Hospitalar Ltda.; Catanduva, SP, Brazil) for 48 hours, washed in running water for 24 hours, and stored in 70% alcohol. Each tibia was scanned with a microtomograph (SkyScan 1272 Bruker MicroCT, Aartselaar, Belgium) in slices of 6 μm (70 Kv e 142 μA), using Al 0.5 mm filter, a rotation step of 0.4 mm, and a pixel resolution of 2016 x 1344 μm, completing the acquisition time of the projections in 1 hour and 36 minutes. The images obtained via projection of X-rays of the samples were stored and reconstituted after the region of interest (ROI) was determined using the NRecon software (Bruker MicroCT, Aartselaar, Belgium) in the following standard configuration: smoothing of 5, correction of rings artifacts of 7, cone beam hardening of 40%, and correction range of images from 0.0 to 0.46. In the DataViewer software (Bruker MicroCT, Aartselaar, Belgium), the images were reconstructed for adjusting the standard positioning for all samples, and observation took place in three planes (transverse, sagittal, and longitudinal). They were oriented in the sagittal plane for the formulation of the dataset. Finally, values for bone-implant contact (region of interest) were analyzed and defined in the CTAn software (Bruker MicroCT, Aartselaar, Belgium) in the following standard configurations: bone volume per tissue volume (BV/TV), trabecular number (Tb.N), trabecular thickness (Tb.Th), and trabecular separation (Tb.Sp).^21,23,24^

### Laser Confocal Microscopy

**Figure 1:**
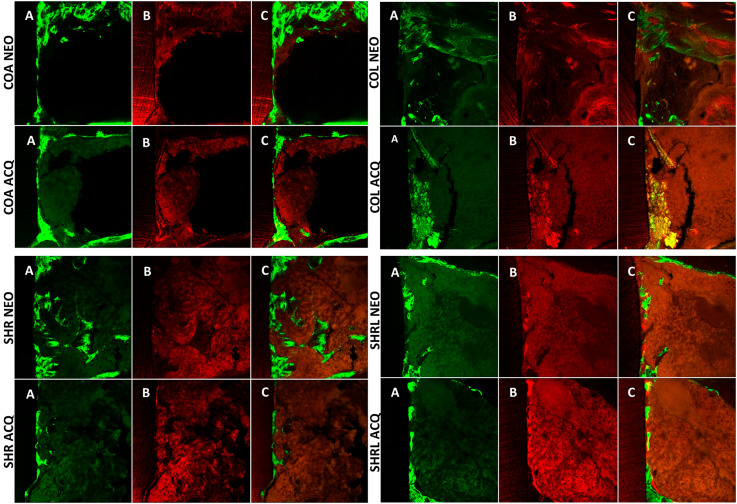
Photomicrographs obtained using Laser Confocal Microscopy for all subgroups: A. Bone area marked with calcein; B. Bone area marked with alizarin; C. Overlapping images of both fluorochromes

After analysis by microcomputerized tomography, the same samples (n=4, per group) were subjected to dehydration with gradually increasing alcohol concentrations. Upon the completion of dehydration, the samples were immersed in a mixture of 100% alcohol and different concentrations of thermo-polymerized methyl methacrylate (JET, Artigos Odontológicos Clássico Ltda.; São Paulo, SP, Brazil). After the process of dehydration of the calcified samples, they were immersed in thermo-polymerized resin (Techno Vit^®^ 7200 VLC, Heraeus Kulzer, GmbH Technical Division, Philipp-Reis-Str. 13/8 D-61273, Wehrheim, Germany) in glass test tubes covered with a lid and were maintained in a laboratory oven at 37°C for five days until the resin was polymerized. The samples were then worn out with a maxicut drill. Subsequently, they were subjected to manual progressive wear by sandpaper of decreasing granulation using Politriz (Arotec S/A Indústria e Comércio, Cotia, SP, Brazil) until slices were 100 μm thick. The slices were mounted on a glass slide with mineral oil (Petrolato Líquido, Mantecor, Taquara, RJ, Brazil) and then had a coverslip placed on top (making sure that no air was trapped) that was sealed with nail polish. With the Leica CTR 4000 CS SPE microscope (Leica Microsystems, Heidelberg, Germany), using 10× objective lens (original increase: 100 times), the longitudinal sections of the bone-implant interface region corresponding to the area of medullary bone tissue were captured, excluding the areas relating to the cortical bone of each tibiae. Therefore, the implant was taken as a reference, and the area of the medullary bone tissue next to the same implant was evaluated in each sample. The images that were captured by the microscope were reconstructed, showing that the medullary bone overlapped the two fluorochromes (calcein and alizarin). These images were transferred to the ImageJ software (Image Processing and Analysis Software, Ontario, ON, Canada). A “color threshold” tool from ImageJ software was utilized in this process, and each image corresponding to the experimental groups was standardized according to hue, saturation, and brightness to show the analyzed fluorochromes. When using the software, the first step was to highlight the green color (calcein) using the tool “measure.” The software showed the corresponding area in μm^2^. The same procedure was used for the color red (alizarin), obtaining data regarding the dynamics of the peri-implant bone tissue. In this methodological approach, the precipitation of new bone was called “active mineralization surface,” represented by alizarin. In order to obtain the mineral apposition rate (MAR - Mineral Apposition Rate) of the fluorochrome area, the images were again transferred to the ImageJ software for the standardization of each image. The tool called “straight” was used, and four lines were drawn between the precipitation at the beginning of alizarin (red marking) up to the end of the precipitation of calcein (green marking). The values were divided by 28, the number of days between the injections of calcein and alizarin, representing the daily precipitation of calcium (Figure 1).^10,23,27,28^ A single blinded and calibrated evaluator performed the laser confocal microscopy analysis. In total, four sections were used per group.

### Statistical analysis

Statistical tests were performed using GraphPad Prism 7.03 (GraphPad software; La Jolla; USA). For the quantitative parameters obtained from biomechanical analysis and mineral apposition rate (MAR from analysis by laser scanning confocal microscopy), normality and homoscedasticity tests (Shapiro-Wilk) were applied in order to verify data distribution. After confirming the normal distribution of the data, the ANOVA test was applied. When necessary, Tukey’s post-test for multiple comparisons was applied in all performed analyses. For all tests, a value of p<0.05 was considered significant.

## Results

### Blood pressure

As expected, the uncontrolled hypertensive animals maintained their high systolic pressure and the losartan-treated SHRs maintained a pattern of hypertension close to that of normotensive animals. There were no systolic changes in the control groups.^9,10,21^

### Immunohistochemical

**Figure 2:**
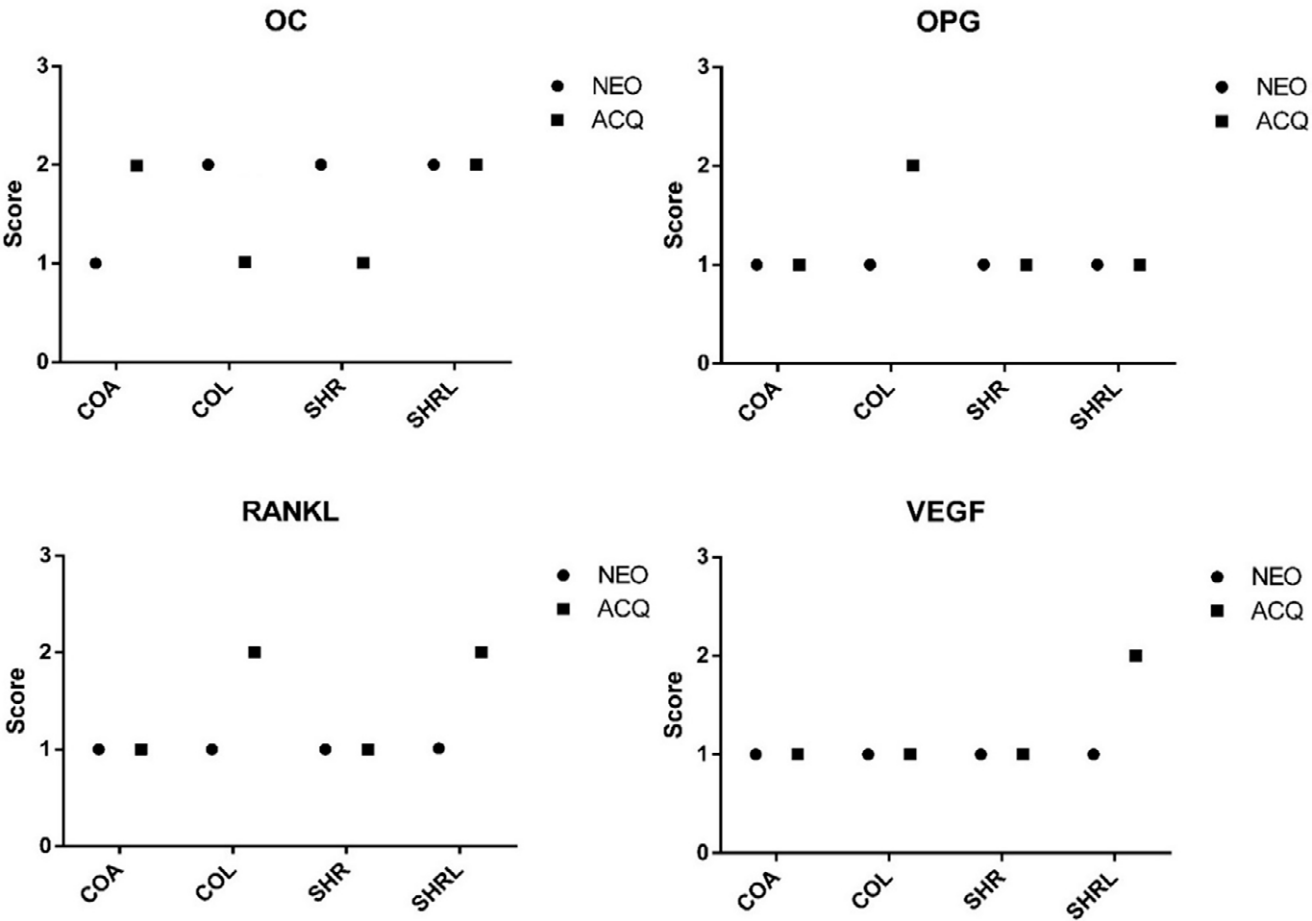
Qualitative analysis of protein expression by scores of the immunohistochemical analysis

After 60 postoperative days, the expression of the OC showed light marking (1) for the subgroups COA NEO, COL ACQ, and SHR ACQ, whereas the expression for the subgroups COA ACQ, COL NEO, SHR NEO, SHRL NEO, and SHRL ACQ was moderate (2), characterizing a better degree of bone tissue mineralization. OPG expression showed light marking (1) for the subgroups COA NEO, COA ACQ, COL NEO, SHR NEO, SHR ACQ, SHRL NEO, and SHRL ACQ. Expression was moderate (2) only for COL ACQ, showing a decrease in osteoclastic action for this subgroup. For RANKL, light marking (1) was shown for the subgroups COA NEO, COA ACQ, COL NEO, SHR NEO, and SHR ACQ. As for the COL ACQ, SHRL NEO, and SHRL ACQ subgroups, expression was moderate (2), demonstrating that the COL ACQ subgroup has a balance in bone remodeling as it obtained a moderate score for OPG. It is also suggested that losartan and the different surface treatments (NeoPoros™ and Acqua™) have no potential against osteoclastogenesis induced by osteoclastic activation by RANKL. Even though the expression of OC for both subgroups has been moderate, it avoided compromising the deposition of the bone matrix. VEGF marking was mild (1) for the subgroups COA NEO, COA ACQ, COL NEO, COL ACQ, SHR NEO, SHR ACQ, and SHRL NEO, expressing itself as moderate (2) only for the SHRL ACQ subgroup. This suggests that both the drug and the hydrophilic (ACQ) surface may provide better angiogenesis to hypertensive bone tissue, improving the blood supply of the peri-implant region for the apposition of the bone matrix, which corroborates with the result found for this subgroup regarding the moderate expression of OC (Figure 2).

### Biomechanical analysis

An increase in the removal torque of the implants of the SHRL ACQ subgroup (10.1 N.cm) was observed when compared to the other groups (p<0.05). Losartan may have favored the negative impact of hypertension for the implant removal torque as the treated control subgroups (COL NEO and COL ACQ) had better values than the absolute control subgroups (COA NEO and COA ACQ) (Table 1).

**Table 1:** Mean of the biomechanical analysis by removal torque (N.cm)

Group	NEO	ACQ
COA	5.9	2.8
COL	6.4	3.3
SHR	9.4 *	4.4
SHRL	5.1	10.1 *

* represents the significant statistical difference (p<0.05) in the different subgroups: SHR NEO x SHR ACQ (p=0.002), SHRL ACQ x COA ACQ (p=0.001), and SHR NEO x COL ACQ

### Microcomputerized tomography

There were no statistically significant differences between the two types of surface treatment. However, the number of trabeculae increased in the treated control group (COL) when compared to the untreated control group (COA) (p<0.05), validating the anabolic effects of losartan on bone tissue as there are greater separation trabeculae for COA (p<0.05) and higher percentage of bone volume for COL on both surfaces (NEO 56.21% and ACQ 54.09%) (Tables 2 to 5 [Table t3] [Table t4] [Table t5]).

**Table 2:** Means of bone volume per tissue volume (BV/TV) (%) by microcomputerized tomography analysis

Group	NEO	ACQ
COA	52.85	53.12
COL	56.21	54.09
SHR	52.60	52.54
SHRL	52.68	51.70

There was no statistically significant difference.

**Table 3 t3:** Means of trabecular number (Tb.N) (mm) by microcomputerized tomography analysis

Group	NEO	ACQ
COA **	3.35	3.35
COL **	3.64	3.52
SHR	3.45	3.34
SHRL **	3.32	3.27

There was no statistically significant difference between subgroups. ** represents the significant statistical difference (p<0.05) for the different groups (control and hypertensive whether or not treated with losartan): COL x COA (p=0.03) and COL x SHRL (p=0.01).

**Table 4 t4:** Means of trabecular thickness (Tb/Th) (mm) by microcomputerized tomography analysis

Group	NEO	ACQ
COA	0.157	0.158
COL	0.154	0.153
SHR	0.152	0.157
SHRL	0.15852	0.158

There was no statistically significant difference.

**Table 5 t5:** Means of trabecular separation (Tb.Sp) (mm) by microcomputerized tomography analysis

Group	NEO	ACQ
COA **	0.159	0.161
COL **	0.151	0.154
SHR	0.154	0.156
SHRL	0.156	0.155

There was no statistically significant difference between subgroups. ** represents the significant statistical difference (p<0.05) for the different groups (control and hypertensive whether or not treated with losartan): COA x COL (p=0.02).

### Laser Confocal Microscopy

As for the active mineralization surface, it was observed that all groups showed a similar average for calcium precipitation for the area marked by the alizarin red fluorochrome. The subgroups COA ACQ (1.35 μm^2^) and SHR ACQ (1.34 μm^2^) showed similar values, suggesting that this surface provides initial postoperative changes in osseointegration (Table 6).

**Table 6:** Means of active mineralization surface (μm2) by laser confocal microscopy

Group	NEO	ACQ
COA	1.10	1.35
COL	1.19	1.11
SHR	1.47	1.34
SHRL	1.48	1.14

There was no statistically significant difference.

There was a statistically significant difference for the different surface treatments (p<0.05) regarding the daily mineral apposition rate (MAR), indicating higher values for the hydrophilic (ACQ) surface in the COA, COL, and SHRL groups (Table 7).

**Table 7:** Means of mineral apposition rates (MAR) (μm/day) by laser confocal microscopy

Group	NEO	ACQ
COA	337.3	580.1*
COL	244.1	661.1*
SHR	487.1	304.6
SHRL	571.5*	588.9*

* represents the significant statistical difference (p<0.05) in the different subgroups considering the comparisons between all subgroups (systemic interference, use of losartan, and implant surfaces): COL ACQ x COL ACQ x COA NEO (p=0.03), COA ACQ x COL NEO (p=0.02), COA ACQ x SHR ACQ (p=0.01), COL ACQ x COL NEO (p=0.002), SHRL NEO x COL NEO (p=0.02), SHRL ACQ x COL NEO (p=0.01), COL ACQ x SHR ACQ (p=0.001), SHRL NEO x SHR ACQ (p=0.01), and SHRL ACQ x SHR ACQ (p=0.009).

## Discussion

Bone healing process has a delay in its chronology in SHR animals, when compared to Wistar rats.^20,22^The question of this study was if different surface treatments could improve peri-implant bone repair in experimental models of bone impaired by treated hypertension whether or not they were treated with the chosen antihypertensive.

Angiotensin II directly acts on the regulation of blood pressure by the renin-angiotensin system. It also indirectly interferes in bone metabolism since it binds to osteoblast AT1 receptors, inciting the expression of RANKL.^4,6,20^ The immunohistochemical findings of this study corroborate the increase in RANKL expression for the hypertensive subgroup treated with losartan and installation of the hydrophilic surface implant (SHRL ACQ), as well as a lower (light) expression for the osteoprotegerin protein (OPG), showing a higher level of bone reabsorption for this subgroup. Both subgroups presented a moderate level of expression for the induction of bone matrix mineralization, represented by the protein osteocalcin (OC), suggesting that losartan and the hydrophilic (ACQ) surface improve the quality of impaired bone tissue since this subgroup has a numerically higher trabecular thickness, confirmed by microcomputerized analysis.

In the control subgroup treated with losartan and installation of the hydrophilic surface implant (COL ACQ), there was a moderate expression for RANKL and OPG, showing a better bone turnover for this subgroup due to the balance between these proteins. Although for COL ACQ, the expression of OC, which represents the formation of the bone matrix, was light, the literature suggests that the hydrophilic surface may be advantageous for peri-implant bone repair, as pointed out in a study that concluded that surface implants hydrophilic (Acqua™) increased BMP-2 protein expression at 45 postoperative days in areas grafted onto rat tibias, besides increased in expression of ALP for this same surface and area at 15 postoperative days.^18^

As for the expression of VEGF, the moderate mark for the SHRL ACQ subgroup explains that the drug treatment associated with the hydrophilic (ACQ) surface may offer an advantage for the osseointegration of implants installed in impaired bone tissue since the increase in local angiogenesis would favor matrix bone formation at the bone-implant interface.

With some heterogeneity from the biomechanical analysis of the data of the control subgroups, both treated and untreated (COA NEO, COA ACQ, COL NEO, and COL ACQ) rough (NEO) and hydrophilic (ACQ) surfaces provide similar implant stability at 60 postoperative days. As for the hypertensive subgroups whether or not treated with losartan (SHR NEO, SHR ACQ, SHRL NEO, and SHRL ACQ), there was a peculiar difference between the different surface treatments proposed by this study. That means that while rough (NEO) surface showed better stability in SHRs, the hydrophilic (ACQ) surface associated with losartan (SHRL ACQ) offered greater and better implant removal torque when compared to all subgroups, corroborating findings in which spontaneously hypertensive rats treated with losartan obtained better results when compared to hypertensive groups and control.^9,10,21^ Another study showed that at 15 postoperative days, the hydrophilic surface provided better biomechanics in the tibias of rats grafted with synthetic material (BoneCeramic - Straumann^®^) to the detriment of the same grafted area with the same material but with the installation of machined surface implants.^18^In contrast, another study using the same surfaces, but with the installation of implants in rabbit tibias, showed that there was no difference in removal torque at 14 and 28 postoperative days.^29^

The data given by microcomputerized tomography revealed no major differences between the surface treatments of the implants, showing that the two types of texturizations show similar responses regarding the microarchitectural characteristics of the reparational bone tissue at 60 postoperative days. Studies done with the same experimental template demonstrated a lower quality of newly formed bone tissue in the area of bone-implant contact in spontaneously hypertensive rats (SHR), as well as the positive effects of losartan on the same area when these animals were treated.^9,10,21^As for the given antihypertensive, there was an improvement in some parameters for the losartan control group (COL NEO and COL ACQ), confirming the favorable effect of its vasodilator action on the peri-implant repair process.^20,21^

In the analysis of results, the hydrophilic (ACQ) surface seems to reverse the imbalance in the bone dynamics process that occurs in hypertensive animals given that combination with losartan promotes a better daily mineral apposition rate (MAR) in comparison to the untreated hypertensive group (SHRL ACQ x SHR ACQ). As shown in a previously mentioned study, alveolar bone turnover in hypertensive animals treated with losartan has better MAR values when compared to hypertensive animals that received no drug treatment.^9,10,21^ Conversely, the data obtained from MAR shows that the best precipitation of mineral ions (calcium and phosphorus) for the hydrophilic (ACQ) surface are independent of the drug in the control subgroups (COA ACQ and COL ACQ), suggesting that this surface treatment may affect the initial phases of postoperative bone mineralization. However, there were no statistically significant differences for the active mineralization surface, which expresses the quantification of new bone. This result seems controversial, but it can be explained due to the time it was studied, in which osseointegration is shown to be complete. Nevertheless, with the data obtained by the microcomputerized tomography analysis in this study, for the parameter “bone volume (BV/TV),” the subgroups COA ACQ and COL ACQ showed higher absolute values when compared to the other subgroups, with the exception of COL NEO, corroborating the findings for MAR.

In this study, the period of evaluation of the stability of the implants was 60 days, in which both surfaces enabled osseointegration. The influence of losartan becomes evident since the quality of bone neoformation can be compensated by the trabecular thickness of treated SHRs in order to compare the bone quality of controls. For the hypertensive subgroup treated with the installation of hydrophilic implants (SHRL ACQ), there was a significant daily mineral apposition rate and peri-implant bone stability in the area, suggesting that there are changes in signaling for osseointegration in early periods of bone remodeling. Also, the subgroups COA ACQ and COL ACQ showed, according to MAR, that the hydrophilic (ACQ) surface, regardless of the drug, can promote changes in the initial stages of bone turnover. That would enable the possible application of immediate loads after the installation of implants with this surface due to the optimization of the beginning of osseointegration, indicating that the wettability and hydrophilicity of this surface may favor bone repair regardless of the drug, as demonstrated in studies with rats, rabbits, dogs, and sheep.^15,17,18,29,30^

## Conclusion

In conclusion, the two implant surfaces presented similar responses regarding the microarchitectural characteristics of the peri-implant bone tissue. Despite the limitations of this study, as well as in the different evaluated systemic conditions, ACQ seemed to improve the initial stages of osseointegration in SHR rats.
